# A Practical Treatise on Sexual Disorders of the Male and Female

**Published:** 1900-09

**Authors:** 


### Book Reviews.

A PRACTICAL TREATISE ON SEXUAL DISORDERS OF THE MALE AND FEMALE. New (2d) edition. By ROBERT W. TAYLOR, M. D , Clinical Professor of Venereal Diseases in the College of Physicians and Surgeons, New York. In one 8vo volume of 435 pages, with 91 illustrations and 13 plates in colors and monochrome. Philadelphia and New York: Lea Brothers & Co. [Price, cloth, $3.00 net.]

The second edition of this work which recently appeared is entirely revised. The author has amplified, added to, and, in places, modified the original text. The subject of sexual disorders in women receives marked attention, several entirely new chapters being added. Many new illustrations appear, and a point very valuable to readers, especially those who know Robert Taylor, is the fact that they are original and can be depended upon as representing existing conditions.

The first four chapters are devoted to the anatomy and physiology of the sexual apparatus, physiology of the male sexual function and the nature and composition of the seminal fluid. The parts are carefully illustrated, there being five colored plates, showing the different anatomical structures in situ. The great help this one feature is to the genito-urinary specialist can scarcely be estimated, not to speak of its value to the general practitioner. The next eight chapters are devoted to a class of diseases and deformities that cause more mental suffering than can be estimated, except possibly by those who are called upon to treat these conditions. If general practitioners would take up and make a careful study of the conditions described by Taylor, applying them as advised, that class of parasites that make their living by treating lost manhood, etc., would soon be driven from the field.

In speaking of curvatures of the penis as a factor in producing organic impotence Taylor refers to over-dilatation and deep cutting of the canal for stricture, as causes of many of these conditions. This is a sound opinion and upon which the author, in our view, should have laid more stress.

In dealing with sterility Taylor brings out a fact that is becoming more apparent every day—namely, that unfruitful marriages are not, as was formerly supposed, always due to the wife, but he states, and his observations are quite correct, that one case in six is due to a condition of sterility in the male. Much stress is laid on epididymitis as a cause, and he could have said with truthfulness that this one complication of gonorrhea may be considered the most important cause of male sterility. The author brings out an important point when he emphasises the continued treatment of this condition until all evidences of induration have disappeared.

In referring to chronic posterior urethritis as a cause of sexual weakness and impotence, Taylor, in mentioning the treatment, sounds the key note when he says "the duration of urethritis has an important bearing upon its treatment." There is no doubt but at least 90 per cent. of the gonorrheal inflammations in this locality would fully resolve, provided irritation by sounds, and the like, was postponed until such time as they might become a necessity for aiding nature. The solutions mentioned and in the strengths advised cannot be improved upon, but the reviewer must disagree with their mode of application. There can be no doubt that the daily or even every-other-day passage of a catheter over an inflamed membrane keeps alive such an inflammation. It must be sterilised every time and unless care is used in holding the meatus so as to balloon the canal, the solution does not come in contact with the whole mucous surface. With the syringe illustrated by the author, the solution can be put into the bladder without any pain, at the same time dilating all rugæ. In the author's reference to the treatment of stricture every point is well taken, dilatation being favored in almost all cases; a most just decision. The complication mentioned might be avoided in nearly all, if not in every case, had he advised thorough cleansing of the canal by an antiseptic solution previous to the passage of any instrument.

Prostatic hypertrophy as a cause of sexual weakness receives attention only so far as a work of this kind will permit, but the few lines in reference to treatment are fully equal to some chapters that occupy considerable space in standard works.

The subject of masturbation in the male is most carefully considered. Taylor claims, and his opinion cannot be disputed, that masturbation if not commenced before the organs have fully developed, and the practice be not carried to excess, will not in a mentally sound person produce much if any damage. He might have referred to the reading of quack literature as being one cause of the nervous symptoms seen in most cases. It would have been well to have advocated home explanations by the parents regarding the outcome of the formation of that loathsome practice, together with that of over indulgence, which is as bad, if not worse, than the former.

The female receives attention to the extent of eleven chapters, Taylor most carefully describing practically the same conditions that have been considered in the male. Certainly this is a valuable work, and it must be conceded that his closing words on spermatorrhea state a fact that should apply not only to that trouble, but all troubles of an imaginary sexual nature—namely, that the subjects of noticeable symptoms of the sexual organs where there has been no previous inflammatory trouble, are weak, and the location of this weakness is in the brain. J. H. D.

